# The role of graphene formed on silver nanowire transparent conductive electrode in ultra-violet light emitting diodes

**DOI:** 10.1038/srep29464

**Published:** 2016-07-08

**Authors:** Tae Hoon Seo, Seula Lee, Kyung Hyun Min, S. Chandramohan, Ah Hyun Park, Gun Hee Lee, Min Park, Eun-Kyung Suh, Myung Jong Kim

**Affiliations:** 1 Applied Quantum Composites Research Center, Korea Institute of Science and Technology, Jeonbuk 565-905, South Korea; 2 School of Semiconductor and Chemical Engineering, Semiconductor Physics Research Center, Chonbuk National University, Jeonju 561-756, South Korea; 3 Photoelectronic Hybrid Research Center, Korea Institute of Science and Technology, Seoul 136-791, South Korea

## Abstract

This paper reports a highly reliable transparent conductive electrode (TCE) that integrates silver nanowires (AgNWs) and high-quality graphene as a protecting layer. Graphene with minimized defects and large graphene domains has been successfully obtained through a facile two-step growth approach. Ultraviolet light emitting diodes (UV-LEDs) were fabricated with AgNWs or hybrid electrodes where AgNWs were combined with two-step grown graphene (A-2GE) or conventional one-step grown graphene (A-1GE). The device performance and reliability of the UV-LEDs with three different electrodes were compared. The A-2GE offered high figure of merit owing to the excellent UV transmittance and reduced sheet resistance. As a consequence, the UV-LEDs made with A-2GE demonstrated reduced forward voltage, enhanced electroluminescence (EL) intensity, and alleviated efficiency droop. The effects of joule heating and UV light illumination on the electrode stability were also studied. The present findings prove superior performance of the A-2GE under high current injection and continuous operation of UV LED, compared to other electrodes. From our observation, the A-2GE would be a reliable TCE for high power UV-LEDs.

Transparent conductive electrodes (TCEs) are essential component of GaN-based light emitting diodes (LEDs), because the LED structure typically contains a *p*-GaN top layer of high sheet resistance and low carrier mobility^1^. Therefore, a cardinal Ohmic contact problem is encountered in the fabrication of LEDs which gives rise to several problems such as severe current crowding near the *p*-electrode, low hole injection efficiency toward active layer, large turn-on voltage, heat generation, and poor device reliability^2^. Indium tin oxide (ITO) has been widely used as a TCE in GaN-based LEDs during the past two decades owing to its high optical transmittance (~90% at 450 nm) and low sheet resistance (as low as 3 ~ 0 Ω/□ at a layer thickness of 150 nm)^3^. Nevertheless, the application of ITO in LEDs is limited due to factors such as high material cost associated with the rapid depletion of indium and sensitivity to acidic and base chemical sources^4^. Moreover, the sharp decline of the optical transparency in the ultraviolet (UV) region with decreasing wavelength seriously limits its application in UV LEDs. Hence, several alternatives, including carbon nanotubes^5^, graphene^6^,^7^,^8^,^9^,^10^,^11^, and metal nanowire^12^,^13^,^14^ have been considerably investigated to substitute ITO. Among the various TCEs, silver nanowires (AgNWs) have become the promising material for application in LEDs owing to its good optical and electrical properties similar or better than those of ITO. A recent study demonstrated that GaN-based LEDs employing AgNWs as TCE could offer low forward voltage and high quantum efficiency at high injection currents, and can exceed the performance of commercial LEDs using either ITO or Ni/Au^12^,^13^. Even though AgNW networks have been successfully applied to LEDs, some problems need to be solved for making this material more durable. For example, the random distribution of AgNWs formed by solution-based coating method gives rise to high resistance for a two-dimensional network and the open spaces between the adjacent nanowires can disturb the vertical current transport toward the active layer. In addition, when AgNWs are directly exposed to air ambient and/or high temperatures of 150 °C caused by joule heating during the high power operation of LEDs, they can be easily oxidized and melted, causing a rapid increase of the sheet resistance and degradation upon long-term use. Hybrid structure of AgNWs and graphene is therefore considered to be a promising alternative to surmount the drawbacks of AgNWs owing to graphene’s outstanding physical properties, such as excellent optical transmittance, high intrinsic mobility, chemical inertness and impermeability to any gases^15^,^16^,^17^,^18^,^19^,^20^,^21^. In this hybrid structure, the graphene on AgNWs serves as a transparent conducting network between adjacent nanowires as well as protective barrier by preventing oxidation of AgNWs, which otherwise could act as current sink nodes. The potential of such hybrid electrode as a TCE of UV-LEDs has been reported in our previous study^18^. According to recent studies, defects and grain boundaries in graphene grown on polycrystalline Cu foil by chemical vapor deposition (CVD) allow transfer of gaseous molecules toward arbitrary surface covered by such graphene layer^22^,^23^,^24^. Thus, although the integration of AgNWs with CVD graphene is simple, achieving a hybrid electrode of high environmental and temperature stability for applications in LEDs requires further breakthrough. For instance, for practical use, it is vital to ensure whole surface coverage by minimizing any structural defects or grain boundaries in graphene.

In this work, a facile two-step growth method is used to synthesize high-quality graphene films. The two-step growth is highly effective in reducing the point defects and increasing the graphene domain size, both factors benefit the graphene to be used as conformal layer to protect the AgNWs from oxidation. Herein, one-dimensional AgNWs network is integrated to graphene synthesized by two-step growth method without any considerable loss in optical transmittance to fabricate exceptionally stable and conductive TCEs. As a proof of concept, we demonstrate operation of a 380 nm UV-LED using this hybrid electrode and compare the performance and long-term reliability with AgNWs only electrode and similar hybrid electrode comprising of AgNWs and conventional one-step grown graphene.

## Results

**Figure 1:**
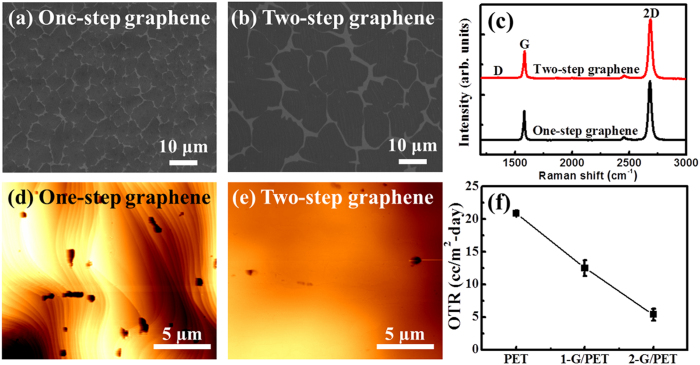
SEM images of early nucleation stage of (**a**) one-step and (**b**) two-step graphene films after a growth time of 60 sec. (**c**) Raman spectra of fully-covered one-step and two-step grown graphene. AFM images of (**d**) one-step and (**e**) two-step graphene on Cu foil subjected to FIFE test. (**f** ) OTR of PET, 1-G/PET, and 2-G/PET, respectively.

In the growth of graphene by CVD, the initial stage of growth is a key to obtain large domain size and good crystal quality. Figure 1(a,b) display scanning electron microscopy (SEM) images of the nucleated graphene after a growth time of 60 sec in the case of one-step and two-step graphene growth processes, respectively. The average domain sizes in one-step and two-step growth are estimated to be approximately 37 and 100 μm^2^, respectively. The corresponding nucleation densities are calculated to be 2.77 × 10^6^/cm^2^ and 5.21 × 10^5^/cm^2^, respectively. Raman spectra were acquired on each graphene films to examine the crystal quality, as shown in Fig. 1(c). Two major peaks i.e., the G-band and the 2D-band are observed in both samples at 1584 cm^−1^ and 2685 cm^−1^, respectively. Raman spectra recorded from both samples reveal typical characteristic of monolayer graphene, i.e. a single Lorentzian peak with a full width at half maximum of 28 cm^−1^ and the 2D to G intensity ratio greater than 2. Moreover, the so-called defects or disorder-provoked D-band peak around 1350 cm^−1^ is negligible, implying that both graphene layers are high quality with minimized defects. Though Raman analysis evaluate the defect distribution and quality of graphene by the D band intensity, it is difficult to detect accurately the low-density defects that unroll across over large area. To give a further insight into structural quality of graphene on large scale, the film-induced frustrated etching (FIFE) test is performed. Commercial copper etchant, ammonium persulfate [(NH_4_)_2_S_2_O_8_], is dropped on to graphene grown on Cu foils. After 10 sec, the dropped etchant is rinsed with deionized water and then the surfaces of one-step and two-step graphene on Cu foils are probed by atomic force microscope (AFM), as shown in Fig. 1(d,e). The etch-pit densities in respective films are estimated to be 1 × 10^7 ^cm^−2^ and 2 × 10^6 ^cm^−2^, signifying that the etch-pit density in two-step graphene is about one order less due to the decrease of the amount of graphene grain boundaries. The reduction of grain boundaries is a result of minimization of nucleation density of graphene during the first growth step and associated decrease in defects caused by better stitching between domain boundaries by fast lateral growth during the second step. To confirm the barrier properties of graphene, the oxygen transmission rate (OTR) measurement is carried out after the graphene films being transferred to poly ethylene terephthalate (PET). The values of OTR for bare PET and PET substrates cover by one-step graphene (1-G/PET) and two-step graphene (2-G/PET) are measured to be 20.89 ± 0.4, 12.52 ± 1.2, 5.39 ± 0.9 cc/m^2^-day, respectively. The 2-G/PET exhibits the lowest OTR value, which is 74% less than that of bare PEF owing to the impermeability of graphene with the minimized defect density and large graphene domain. It is evident that graphene plays an important role as gas barrier against oxygen.

**Figure 2:**
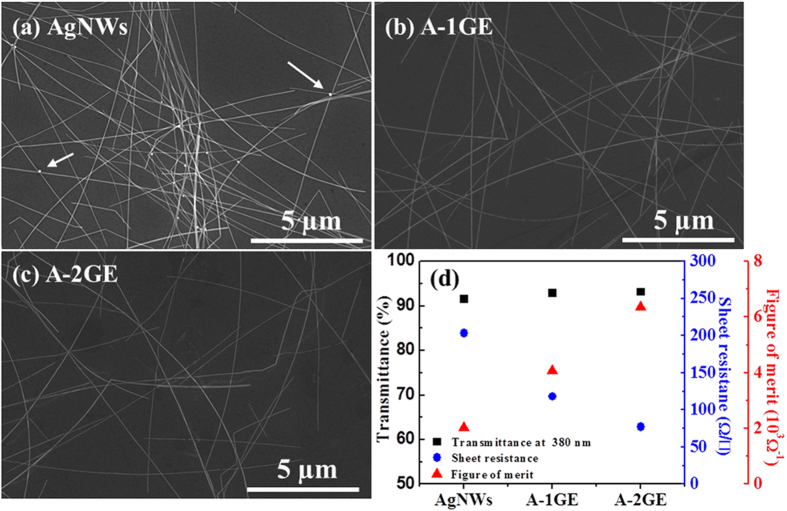
SEM images of (**a**) AgNWs, (**b**) A-1GE, and (**c**) A-2GE formed on sapphire substrate. (**d**) Optical transmittance, sheet resistance, and figure of merit of three different TCEs studied.

Figure 2 shows SEM images of AgNWs only electrode (Fig. 2a) and hybrid electrodes of AgNWs-graphene combination formed by using one-step graphene (Fig. 2b) and two-step graphene (Fig. 2c) on sapphire substrate. It may be noted that the AgNWs used in this work have an average diameter of approximately 35 nm (see Fig. S2 in the Supporting Information) and an average length of few tens of micrometers, and formed random percolation networks without severe aggregation. The white arrows in Fig. 2(a) represent formation of silver oxides within the AgNWs, which could influence carrier transport across nanowire junctions. This will be addressed later when sheet resistance results are discussed. However, SEM images of AgNWs covered by either graphene did not show presence of any silver oxides in the vicinities of the nanowire junctions, because unfavorable gas or molecules cannot transfer via the graphene layer toward AgNWs, as discussed earlier with reference to Fig. 2(b,c). SEM images further show that the suppleness of graphene allows it to encompass the nanowire junctions like a plastic wrap.

**Figure 3:**
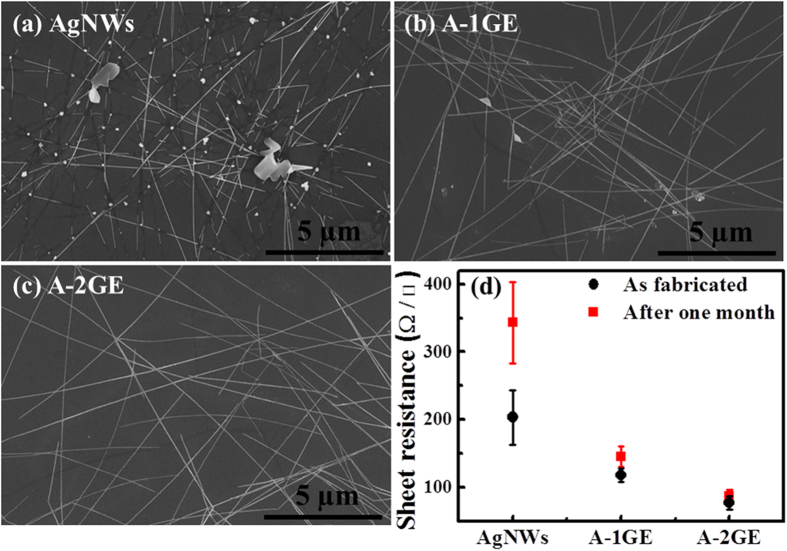
(**a**–**c**) SEM images and (**d**) sheet resistance of AgNWs, A-1GE, and A-2GE after one month period.

Optical transparency and sheet resistance are the two important criterias that must be considered for the use of TCEs in LEDs, because the efficiency of the LEDs largely associated with these two parameters. The transmittance, sheet resistance, and figure of merit (ϕ_TC_) of the three different TCEs are depicted in Fig. 2(d). The figure of merit, defined by the Haacke equation as ϕ_TC_ = T^10^/R_sh_^25^, is calculated using the sheet resistance (R_sh_) and transmittance measured at 380 nm. The transmittance of the AgNWs, A-1GE, and A-2GE at 380 nm is measured to be 91.5%, 93%, and 93%, respectively. Generally, some loss in optical transmittance could occur in hybrid TCEs due to the additional absorption by graphene in the visible wavelength region. Conversely, the transmittances of A-1GE and A-2GE at 380 nm are increased about 1.5% compared to the transmittance of AgNWs (91.5%), which is a likely result of partial screening of plasmon absorption of AgNWs. This is because the dielectric environment of the AgNWs is interrupted by the graphene, which inhibits the collective oscillation of free electrons in AgNWs arising due to interacting electromagnetic field^26^^,^^27^. The change in sheet resistance after the integration of AgNWs with graphene is also investigated. The sheet resistance measurements were performed on a Hall Effect measurement system by defining Van der Pauw contacts at room temperature. The measured sheet resistance values of AgNWs, A-1GE, and A-2GE electrodes are 205.1 ± 40 Ω/□, 117 ± 10 Ω/□, and 77.5 ± 10 Ω/□, respectively. The reduced sheet resistance observed for the hybrid electrode is due to the added new conduction paths by bridging AgNWs or grain boundaries in graphene^28^,^29^, each complementing the disadvantages of the other component. Also, the sheet resistance of the A-2GE is relatively less compared to the A-1GE, which is due to the fact that the two-step graphene has relatively low defects and large domain size compared to one-step graphene, as discussed previously. The A-2GE offers best ϕ_TC_ due to the reduced sheet resistance and increase in transmittance at 380 nm, as shown in Fig. 2(d). The main advantage of A-2GE can be realized if one considers the properties of the electrodes after long-time exposure to the ambient. Figure 3(a–c) show SEM images of the three electrodes after being exposed to ambient for one month. In the case of AgNWs only electrode, oxidation of silver nanowires and nanowire-nanowire junction breakdown caused by oxidation are evident from Fig. 3(a). One can see that enough nanowires are broken up, leading to increased sheet resistance of 343.2 ± 60 Ω/□, as shown in Fig. 3(d). In the case of A-1GE, small or large clusters of silver oxides are observed on the surface of nanowires, whereas the SEM image of A-2GE show formation of negligible silver oxides due to the impermeable nature of two-step graphene governed by the low defect density and large size graphene domains. Accordingly, the sheet resistance of the A-2GE remains almost unchanged at a value of 86.6 ± 10 Ω/□ when measured after one month. It is noteworthy that the sheet resistance of the A-1GE after one month is relatively higher (145.3 ± 15 Ω/□) compared to A-2GE and the AgNWs in A-1GE are partially oxidized at junctions between nanowires. This result further confirms that the two-step graphene is highly impermeable to any gases compared to one-step graphene.

**Figure 4:**
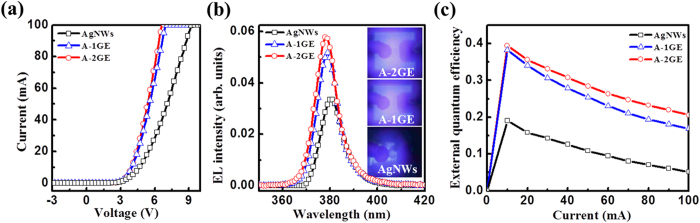
(**a**) Current-voltage (I–V) curves, (**b**) EL spectra at an injection current of 20 mA, and (**c**) EQE of UV-LEDs with AgNWs, A-1GE, and A-2GE as TCEs. (**b**, inset) EL images taken at 20 mA.

The performances of AgNWs, A-1GE, and A-2GE as TCEs in UV LEDs of 380 nm emission wavelength are evaluated. Figure 4(a,b) illustrate the I–V characteristics and electroluminescence (EL) of the fabricated UV-LEDs with three electrodes, respectively. Forward voltages at an injection current of 20 mA are measured to be 5.5, 4.5, and 4.4 V for the UV-LEDs having AgNWs, A-1GE, and A-2GE, respectively. The relatively high forward voltage involved with AgNWs only electrode is attributed to the high sheet resistance. When graphene barrier layer is introduced, the I–V curves become more linear with a considerable reduction in the forward voltage. This result indicates that the sheet resistance influences the series resistance of the diode. Considering this fact, the low series resistance and forward voltage observed in devices with A-2GE suggest a better TCE performance compared to A-1GE due to the relatively low sheet resistance of A-2GE (See Fig. S4(a) in the Supporting Information). That is, the two-step graphene as a barrier layer could act as efficient lateral current diffusion pathways for AgNWs which then inject current to the active junctions of the LED via *p*-GaN layer. As a result, the electroluminescence (EL) intensity is enhanced for the LED with A-2GE in comparison to LEDs made with AgNWs or A-1GE, as shown in Fig. 3(b). The EL peak position of the LED with A-2GE also exhibits a slight blue-shift compared to devices made with other electrodes. This can be attributed to the combined factors of increased band-filing caused by more carrier injection towards the active junction and reduced heating effect offered by relatively low series resistance^13^. This effect can be further understood from the EL images, which show the light emission is non-uniform and bright near the *p*-electrode for the LED with AgNWs due to insufficient current spreading by the formation of loosely bound random networks of AgNWs. Of particular point of interest is that a blue LED fabricated with similar AgNWs only electrode offered well-distributed current over the whole emission area, as shown in Fig. S5(a) of the Supporting Information. This difference can be attributed to the fact that the crystal quality of the epitaxial layers differs in respective LED structures which could affect the current spreading in the device. In general, the crystal quality of the epitaxial layers of UV-LED structure is naturally inferior to those of blue LED due to the more resistive *p*-GaN/*p*-AlGaN layers in the former, and hence it will have an additional influence on current spreading and light output. However, recent studies demonstrated uniform light emission over the entire area of an UV-LED with the application of AgNWs TCE^12^,^13^. The better performance of the nanowire electrode reported by others can be attributed to relatively low sheet resistance, which in their experiment was 11.7 Ω/□ or 30 Ω/□, whereas the sheet resistance of the AgNWs electrode used in our experiment is only 205 Ω/□. In other words, the density of nanowires forming the electrode is significantly less in our structure, compared to the previous cases. Thus, significantly reduced contact area can be expected at the nanowire/p-GaN interface in our device, which might contribute to large current crowing. In addition, previous approaches involved use of commercial quality wafer^12^ and thermal annealing to reduce the contact resistance^13^, both factors further restrict a direct comparison of the present results. Even though the performance of the AgNWs electrode is not superior, the better performance of the hybrid electrode using two-step graphene (A-2GE) in UV-LED signifies that it supplication will be far more central in large-chip device that have long current spreading lengths. The external quantum efficiencies (EQE) of the LEDs are obtained from the integrated EL intensity and its variation with injection current is shown in Fig. 4(c). All the three devices exhibit typical efficiency droop behavior, a decrease in the EQE with increasing current; the EQE increases rapidly at injection currents of 10 mA, begins to roll off, and starts to decrease as current increases. The EQE droop, defined as (EQE_max_-EQE)/EQE_max_ is found to be 72%, 55.8%, and 47% for UV-LEDs with AgNWs, A-1GE, and A-2GE, respectively, at an injection current of 100 mA. The primary reasons for the reduced efficiency droop associated with the use of A-2GE are decrease in the heating effect caused by the reduced series resistance and improved current spreading^30^,^31^.

## Discussion

**Figure 5:**
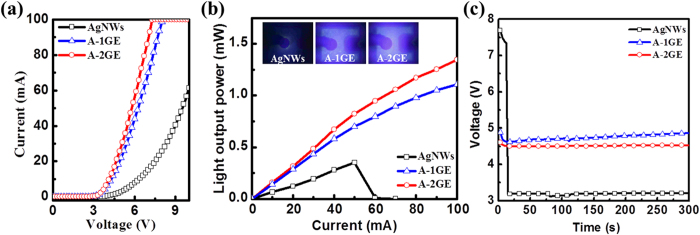
(**a**) I–V curve and (**b**) light output power with increasing current for UV-LED having a variety of TCEs investigated in this work after one month. (**b**, inset) EL photographs of respective devices at 20 mA. (**c**) Voltage values with increasing time for UV-LEDs with three different electrodes under 20 mA continuous current injection.

For practical application of TCE, it is important to take into account the long-term stability and reliability of TCE material. Herein, we intend to compare the performance of all the three devices after one month. Figure 5(a,b) show the I–V and light output power characteristics of the UV-LEDs with various TCEs as a function of injection current after an aging period of one month. When the injection current is 20 mA, the forward voltages are found to be 7.6, 4.9, and 4.6 V for LEDs having AgNWs, A-1GE, and A-2GE, respectively. The forward voltage of the LED using AgNWs is significantly increased compared to the value measured initially, indicating that AgNWs are unstable because AgNWs have a tendency to oxidize when exposed to environment over long period of time, as show in Fig. 3(a). On the other hand, the forward voltages of the LEDs with hybrid electrodes show marginal increase from 4.5 V to 4.9 V and 4.4 V to 4.6 V for the devices with A-1GE and A-2GE, respectively. This result suggests that graphene servers as a protecting layer for the underlying AgNWs against gaseous molecules owing to its impermeable property. One can notice that the variations in the forward voltage and series resistance of the device with A-2GE after one month are marginal when compared to the device with A-1GE, as shown in Fig. 5(a) and Fig. S4(b) of the Supporting Information. These results indicate that two-step graphene acts as an excellent gas-barrier and protection layer than one-step graphene due to the reduced defect densities and increase of graphene domain size. To further support our conclusion, the light output power as a function of injection current for the UV-LEDs with three different TCEs examined in Fig. 5(a) is illustrated in Fig. 5(b). The light output power of the LED with AgNWs is poor and no light emission is observed when the injection current exceeded 40 mA because of device failure caused by severe voltage drop and junction breakdown. When operated at high injection current levels, the device inevitably generates large heat, obstructing the reliability and performance of the device. According to Fig. 5(b), the UV-LEDs with A-1GE and A-2GE show stable operation even after one month up to an injection current of 100 mA with bright light emission over the entire area, as evidenced from the respective EL images of the device at 20 mA. This result strongly implies that both devices with graphene have thermal stability during long-time operation. One can notice that the difference in light output power between the two devices with A-1GE and A-2GE progressively increases with increasing injection current. The higher light output observed for the LED with A-2GE suggests that the A-2GE as a TCE maintains great thermal as well as ambient stability and hence offers device reliability better than that of the device made use of A-1GE.

Meanwhile, studies have shown that moderate UV exposure could lead to degradation of the performance of AgNWs^32^,^33^. Therefore, the stability of the AgNWs in UV LEDs due to self-illumination is also an important issue need to be taken into consideration. To investigate this effect and understand the stability of the electrodes when exposed to 380 nm light emission for long-time, the forward voltages of each LED are measured as a function of time under continuous current injection at 20 mA, as shown in Fig. 5(c). One can notice that the voltage drops to zero for the device with AgNWs within a short time of 12 sec, because the AgNWs crumbles due to self-heating, forming small segments or metal beads during the continuous current injection (See Fig. S6(a) in the Supporting Information). In contrast, devices using hybrid electrode are capable of operating for a longer period of time, which is evident from the measurements where the devices are tested up to 300 sec. Thus, it is obvious that hybrid electrodes with graphene conserve the nanowire geometry or property during the UV emission, as shown in Fig. S6(b,c) of the Supporting Information. Note that the voltage of the UV-LED with A-1GE gradually increases when the operation time increases due to the degradation in the electrical properties caused by an initial damage through point defects or grain boundaries of one-step graphene. This can be seen as white lines in the SEM image shown in Fig. S6(b) in the Supporting Information. On the other hand, the LED with A-2GE showed more stability with almost constant voltage under continuous current injection up to 300 s. This is attributed to the better quality of the two-step graphene where the fast lateral growth facilitated by the high carbon injection rate allows strong binding between domain boundaries and hence defects or grain boundaries are greatly suppressed as evident from the SEM images (Fig. S6(c)). Based on the present findings, the two-step graphene as an effective heat spreading barrier layer plays an important role in shielding the AgNWs from degradation by any joule heating, oxidation, and UV exposure.

In summary, the impact of the graphene quality on the performance of a hybrid electrode of graphene on Ag NWs in GaN-based UV light-emitting diodes has been studied. Two dissimilar graphene films grown by one-step and two-step approaches were evaluated as a protective transparent conductive coating to Ag NWs towards the fabrication of extremely stable TCEs for UV-LEDs. The hybrid electrode using two-step graphene showed good ambient stability with stable sheet resistance over time. The UV LED using this TCE offered a low forward voltage, an increase in the EL intensity, and a reduction of efficiency droop. Furthermore, the device exhibited stable light emission even at high injection currents while the Ag NWs only electrode degraded over time. Our findings suggest that high-quality graphene on Ag NWs as hybrid TCE has great potential in high-power devices owing to its stability and reliability.

## Methods

### Silver nanowires

The AgNWs used in this work was purchased from NANOPYXIS Corp. and diluted to a concentration of 5 mg/ml before being used. The diluted aqueous dispersion was then spin-coated on to desired substrates at 1000 rpm for 40 seconds to form the transparent electrodes.

### Preparation of hybrid electrodes

We prepared three different TCEs as follows: (1) a bare AgNWs electrode, (2) a hybrid electrode of AgNWs-graphene synthesized by conventional one-step growth (A-1GE) and (3) a hybrid electrode of AgNWs-graphene synthesized by two-step growth (A-2GE). Firstly, an aqueous solution containing AgNWs was spin-coated on substrates of interest at 1000 rpm for 40 s, which is the optimum condition in terms of transmittance-sheet resistance tradeoff and more details can be found in ref. 34. Graphene layer investigated in our work was synthesized on 35-μm-thick Cu foils (Nippon Mining) by low pressure chemical vapor deposition (LPCVD). The Cu foils were placed in a 4 inch quartz tube and gradually heated up to 1030 °C for 1 h under a H_2_ flow rate of 15 standard cubic centimeters per minute (sccm) by split-tube furnace. Concurrently, the CVD chamber was pumped down to 0.072 Torr. Then, the Cu foil was annealed for 50 min. In the one-step growth, graphene synthesis was carried out under flowing CH_4_ of 13 sccm and H_2_ of 15 sccm at 1030 °C for 23 min. In the case of two-step growth, large size graphene domains were initially achieved on Cu foils by reducing the nucleation sites in a mixed CH_4_/H_2_ (5 sccm/100 sccm) ambient for 1 min at 1030 °C. In the second step, continuous graphene was obtained when the flow rate of CH_4_ and growth time were increased to 13 sccm and 8 min, respectively, while maintaining the H_2_ flow rate and temperature constant. Finally, the chamber was cooled down to room temperature by injecting 500 sccm of Ar. Polymethyl methacrylate (PMMA) was spin-coated onto graphene surface at 4200 rpm for 50 s to make a supporting layer for the graphene during the transfer to other substrates of interest. Thereafter, the Cu foil with PMMA-spun graphene was immersed in 0.1M Ammonium Persulfate [(NH_4_)_2_S_2_O_8_] solution for 4 h to remove the Cu foil. PMMA/graphene layer was transferred to AgNWs-coated substrates in order to form the hybrid electrode. Finally, the PMMA was removed by using acetone. More details on the growth of graphene and transfer can be found in ref. 35.

### Growth and fabrication of UV-LEDs

The UV LED structure used in this study is composed of an un-doped GaN layer, a Si-doped *n*-type GaN layer, five-pairs of In_0.04_Ga_0.96_N/Al_0.08_Ga_0.02_N MQWs, a Mg-doped *p*-Al_0.25_Ga_0.75_N electron blocking layer, and a *p*-type GaN cladding layer. Briefly, the Si-doped *n*-GaN layer of 2-μm was grown on un-doped GaN layer at 1100 °C under a pressure of 400 mbar for 60 min by metal organic chemical vapor deposition (MOCVD). Subsequently, five pairs of InGaN quantum wells and GaN barrier layers of thickness 3 nm and 12 nm, respectively, were grown at 790 °C and 810 °C, to form the active region. Then, a 25 nm-thick Mg-doped *p*-Al_0.25_Ga_0.75_N electron blocking layer and a 100 nm-thick *p*-GaN contact layer were grown at 1040 °C. Following the growth of LED epi-wafer, discrete LED devices were fabricated with a chip size of 350 × 350 μm^2^ in which the mesa region was defined by an inductively coupled plasma reactive ion etching system using Cl_2_/BCl_3_ gases until *n*-GaN layer was revealed for *n*-electrode contact. Subsequently, different TCEs were formed on *p*-GaN layer as explained in the previous section. As a final step, Cr (50 nm)/Au (250 nm) metals for the *n*- and the *p*-electrode were deposited onto both the transparent electrode and the *n*-GaN layer using electron beam evaporator.

### Characterization

Field emission scanning electron microscopy (FESEM, NovaSEM 450) was used to probe the surface morphology of the samples investigated in this work. Optical transmittance and sheet resistance measurements were performed on a UV/VIS spectrometer (V-670EX) and a four-point probe system (CMT-SR1000N), respectively. The surface topography of graphene on Cu after the film-induced frustrated etching (FIFE) test was probed by atomic force microscope (AFM, Park NX10) in tapping mode. The quality of graphene was characterized by Raman spectroscopy using 514 nm-line of an Ar ion laser as an excitation source. Current-Voltage (I–V) and electroluminescence (EL) measurements on the LED devices were performed using a probe station system. The oxygen transmission rate (OTR) was analyzed by a commercial MOCON instrument.

## Additional Information

**How to cite this article**: Seo, T. H. *et al*. The role of graphene formed on silver nanowire transparent conductive electrode in ultra-violet light emitting diodes. *Sci. Rep.* **6**, 29464; doi: 10.1038/srep29464 (2016).

## Supplementary Material

Supplementary Information
