# Supplementary material for: The role of graphene formed on silver nanowire transparent conductive electrode in ultra-violet light emitting diodes
Source: Sci Rep. 2016 Jul 8;6:29464. doi: 10.1038/srep29464 (PMC4937441; doi:10.1038/srep29464)
Supplement: Supplementary Information [file srep29464-s1.pdf]

The role of graphene formed on silver nanowire transparent conductive electrode in ultra-violet light emitting diodes

Tae Hoon Seo,[1] Seula Lee,[1] Kyung Hyun Min,[1,3] S. Chandramohan,[3] Ah Hyun Park,[3] Gun Hee Lee,[3] Min Park,[2] Eun-Kyung Suh [3,] and Myung Jong Kim [1,*]*

[1] Applied Quantum Composites Research Center, [2] Photoelectronic Hybrid Research Center, Korea Institute of Science and Technology, Jeonbuk 565-905† and Seoul 136-791‡, South Korea

[3] School of Semiconductor and Chemical Engineering, Semiconductor Physics Research Center, Chonbuk National University, Jeonju 561-756, South Korea

* **Corresponding author**. Tel.: +82-63-219-8135, +82-63-270-3441

E-mail: myung@kist.re.kr (M. J. Kim)

E-mail: eksuh@jbnu.ac.kr (E.-K. Suh)

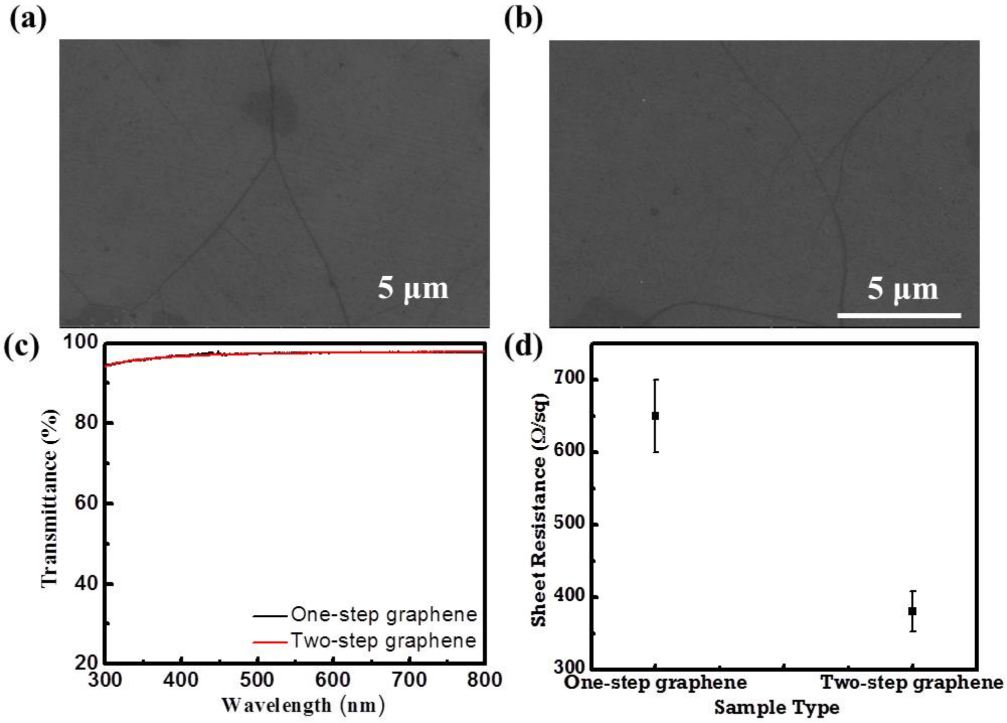

Figure S1. SEM images of fully-covered (a) one-step and (b) two-step graphene, respectively. (c) and (d) their opto-electrical properties.

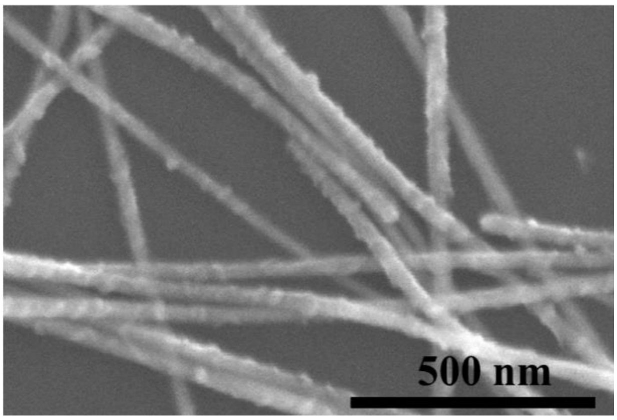

Figure S2. The SEM image of AgNWs formed on sapphire.

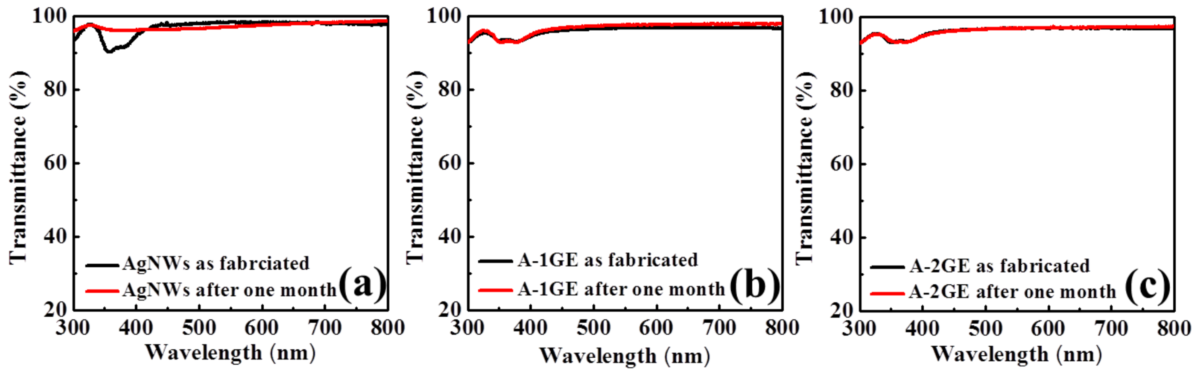

Figure S3. Transmittance characteristics of (a) AgNWs, (b) A-1GE, and (c) A-2GE as a function of wavelength on polished sapphire substrate at initial and after one month.

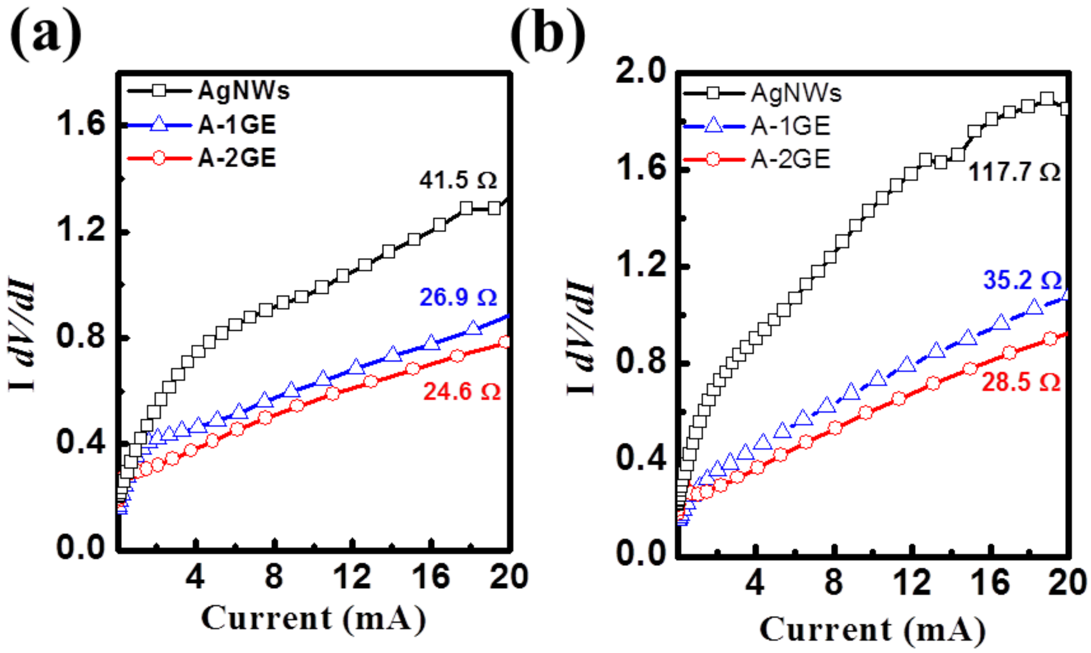

Figure S4. I(*dV/dI*) versus I plots for LED with various electrodes at (a) initial and (b) after one month

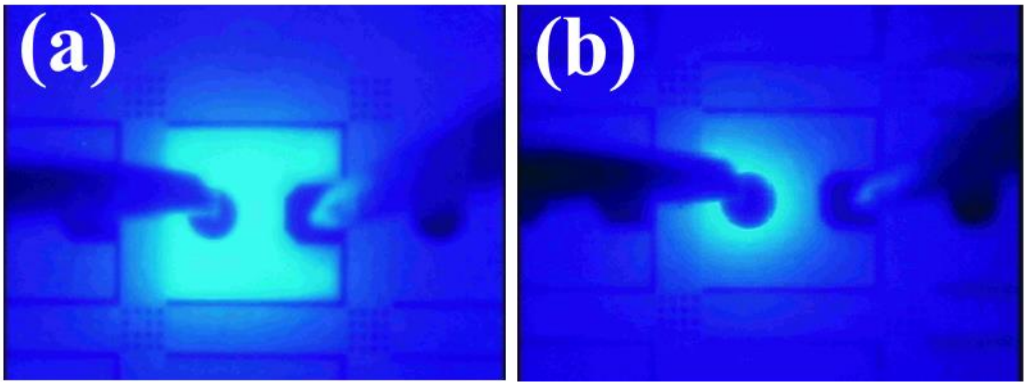

Figure S5. EL images of blue-LEDs using bare AgNWs during light emission at an injection current 5 mA at (a) first and (b) after one month.

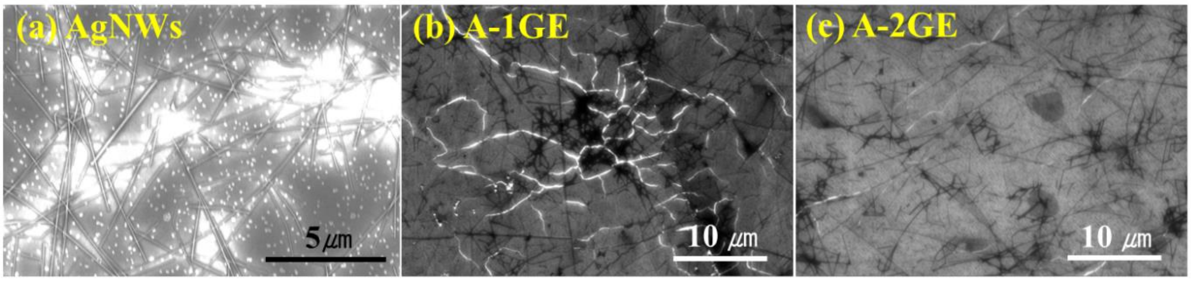

Figure S6. SEM images of (a) AgNWs, (b) A-1GE, and (c) A-2GE after exposed 380 nm UV emission during 300 s, respectively.
